# Genetic Dissection of Internode Length Above the Uppermost Ear in Four RIL Populations of Maize (*Zea mays* L.)

**DOI:** 10.1534/g3.114.016378

**Published:** 2014-12-23

**Authors:** Lixia Ku, Liru Cao, Xiaomin Wei, Huihui Su, Zhiqiang Tian, Shulei Guo, Liangkun Zhang, Zhenzhen Ren, Xiaobo Wang, Yuguang Zhu, Guohui Li, Zhiyong Wang, Yanhui Chen

**Affiliations:** * College of Agronomy, Synergetic Innovation Center of Henan Grain Crops and National Key Laboratory of Wheat and Maize Crop Science, Henan Agricultural University, Zhengzhou, 450002, China; † Henan University of Animal Husbandry and Economy, Zhengzhou, 450046, China

**Keywords:** maize, RIL population, internode length, QTL mapping, major QTL

## Abstract

The internode length above the uppermost ear (ILAU) is an important influencing factor for canopy architecture in maize. Analyzing the genetic characteristics of internode length is critical for improving plant population structure and increasing photosynthetic efficiency. However, the genetic control of ILAU has not been determined. In this study, quantitative trait loci (QTL) for internode length at five positions above the uppermost ear were identified using four sets of recombinant inbred line (RIL) populations in three environments. Genetic maps and initial QTL were integrated using meta-analyses across the four populations. Seventy QTL were identified: 16 in population 1; 14 in population 2; 25 in population 3; and 15 in population 4. Individual effects ranged from 5.36% to 26.85% of phenotypic variation, with 27 QTL >10%. In addition, the following common QTL were identified across two populations: one common QTL for the internode length of all five positions; one common QTL for the internode length of three positions; and one common QTL for the internode length of one position. In addition, four common QTL for the internode length of four positions were identified in one population. The results indicated that the ILAU at different positions above the uppermost ear could be affected by one or several of the same QTL. The traits may also be regulated by many different QTL. Of the 70 initial QTL, 46 were integrated in 14 meta-QTL (mQTLs) by meta-analysis, and 17 of the 27 initial QTL with R^2^ >10% were integrated in 7 mQTLs. Four of the key mQTLs (mQTL2-2, mQTL3-2, mQTL5-1, mQTL5-2, and mQTL9) in which the initial QTL displayed R^2^ >10% included four to 11 initial QTL for an internode length of four to five positions from one or two populations. These results may provide useful information for marker-assisted selection to improve canopy architecture.

Maize is a globally important food and feed crop, and improving maize yield per unit area is a major goal of maize breeding. Plant architecture breeding to improve the plant type and photosynthetic efficiency per unit area is a primary approach for achieving high yield in maize. Plant architecture influences solar radiation capture within the canopy, nitrogen reservoirs for grain filling, abiotic stress tolerance, and biological and economic output per unit area. By selecting leaf architecture, root architecture, stem architecture, and ear architecture, among others, an ideal plant architecture can be created to allow maize to fully capture and use solar energy at all growth stages and maximize maize yields per unit area. The remarkable importance of plant architecture in maize is clear when hybrids are retrospectively analyzed (Duvick 1977, 2005; Duvick and Cassman1999; Russell 1985, 1991; Sakamoto *et al.* 2006; Tollenaar and Wu 1999; Troyer 2001). The genetic controls underlying stem architecture are of particular interest for maize plant architecture improvement. The stem architecture includes the plant height, ear height, stem diameter, and internode length. Plant and ear heights have been studied extensively (Austin *et al.* 2001; Beavis *et al.* 1991; Berke and Rocheford 1995; Koester *et al.* 1993; Li *et al.* 2007); however, limited studies have been conducted on internode length, which is one of the decisive factors affecting plant and ear height. In grass, internode length is attributed to the development of intercalary meristems at the base of the growing internode, which is capable of cell division and cell elongation (Sauter and Kende 1992; Van Der Knaap *et al.* 2000). Internode length in maize, particularly internode length above the uppermost ear (ILAU), is an important index that reflects optimal canopy architecture. Therefore, establishing equitable ILAU is critical for improving plant architecture, population quality, photosynthetic efficiency, and grain yield.

In maize, internode length affects lodging resistance as well as yield per unit area. Shortening the internode length below the ear enhances resistance to lodging (Cao *et al.* 2006; Lü *et al.* 2008). Increasing three consecutive internode lengths (the first leaf above the uppermost ear, the leaf of the ear, and the first leaf below the ear) improves female spike differentiation, resulting in larger ears and increased grain weight (Cao *et al.* 2006). Increasing the ILAU decreases the canopy height, improves ventilation and light penetration, and enhances photosynthesis in the middle and lower leaves as well as the efficiency of light energy utilization for the entire plant (Guo *et al.* 2005). In general, as the ILAU increases, the middle and upper leaves capture more light and conductive photosynthetic activity is enhanced.

With the rapid development of high-density molecular marker linkage maps and quantitative trait loci (QTL) detection approaches, a large amount of data are currently available for maize QTL, such as yield, plant height, ear height, and resistance. However, QTL studies of ILAU have not been performed, and the genetic basis of ILAU remains unclear. To dissect the genetic basis for ILAU in maize, QTL were mapped using four sets of recombinant inbred line (RIL) populations derived from crosses (Yu82×Yu87-1, Yu82×Shen137, Yu87-1×Zong3, and Yu537×Shen137). The objectives were to identify additional genomic regions of QTL for internode length above the uppermost ear, to estimate the magnitude and type of their genetic effects, and to provide information for fine mapping and marker-assisted selection.

## Materials and Methods

### Population development

The four sets of study populations consisted of 208, 197, 223, and 212 RILs derived from the crosses Yu82×Yu87-1, Yu82×Shen137, Yu87-1×Zong3, and Yu537A×Shen137, respectively. The four populations were designated Population 1 (Pop. 1), Population 2 (Pop. 2), Population 3 (Pop. 3), and Population 4 (Pop. 4).

The parents of four populations were chosen based on distinct maize germplasm groups. Inbred lines Yu82, Yu537A, and Zong3 were derived from a Chinese Stiff Stalk germplasm, which is a heterotic group used broadly in China, whereas inbred lines Shen137 and Yu87-1 were derived from a non-Stiff Stalk germplasm, which is also a heterotic group used broadly in China.

### Field trial and trait evaluation

**Figure 1:**
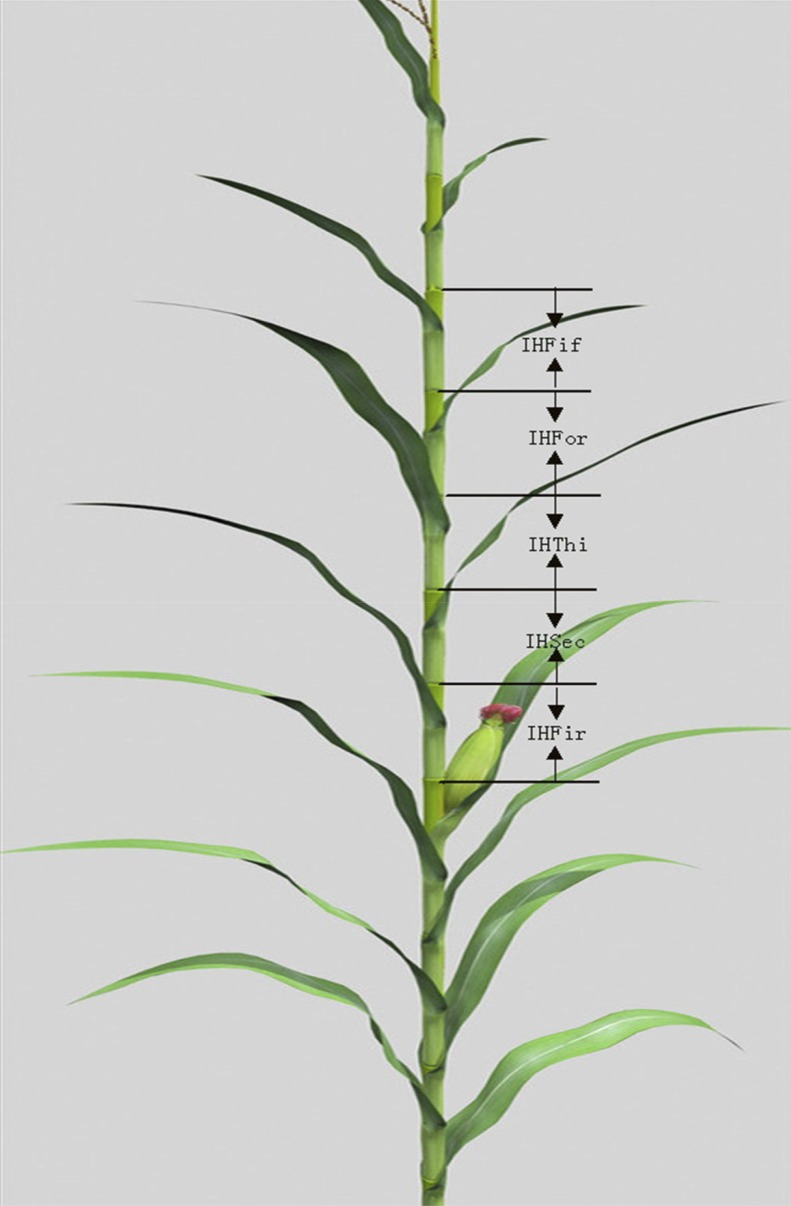
Schematic explanation of ILFir, ILSec, ILThi, ILFor, and ILFif. IL indicates the length from node to node; ILFir, the first internode length above the uppermost ear; ILSec, the second internode length above the uppermost ear; ILThi, the third internode length above the uppermost ear; ILFor, the fourth internode length above the uppermost ear; and ILFif, the fifth internode length above the uppermost ear.

The four populations of five parents were evaluated in three environments in 2011 and 2012: Pop. 1 was planted in Zhengzhou, Anyang, and Zhumadian of Henan Province in 2011; Pop. 2 was planted in Shangqiu, Wenxian, and Nanyang of Henan Province in 2011; Pop. 3 was planted in Zhengzhou, Puyang, and Shangqiu of Henan Province in 2012; and Pop. 4 was planted in Wenxian, Nanyang, and Anyang of Henan Province in 2012. Each field experiment followed a randomized complete block design with three replicates. Each plot included one row that was 4 m long and 0.67 m wide and had a total of 17 plants at a density of 60,000 plants/ha. Ten days after pollen shed, five consecutive plants from the middle of each plot were chosen to evaluate the ILAU (ILFir, ILSec, ILThi, ILFor, and ILFif, the first, second, third, fourth, and fifth internode length above the uppermost ear, respectively), and each internode length was defined as the length from node to node (Figure 1). Family trait values were reported as the average from five plants in each replicate. The overall performance was determined by calculating the average over the three experimental environments. Descriptive statistics and simple Pearson correlation coefficients (r) were calculated between traits using SPSS 12.0 software (SPSS Inc., Chicago, IL).

### Molecular linkage construction and QTL analysis

Young leaf samples at the seedling stage from the four RIL populations were collected, and genomic DNA was extracted using the cetyltrimethylammonium bromide (CTAB) method (Saghai-Maroof *et al.* 1984). The genotype data for each single nucleotide polymorphism (SNP) marker were analyzed using maize SNP3K (Illumina Inc., San Diego, CA), which generated homozygous and heterozygous genotype clusters. A total of 3072 SNP markers were selected as per the manufacturer’s recommendation to analyze the four populations and corresponding parents. Ultimately, the four linkage maps were created using Joinmap version 4.0 software and consisted of 1179, 1116, 1243, and 1102 SNP markers. The parameters included a logarithm of odds (LOD) threshold >3.0, total length of 1873.02 cM for Pop. 1 with an average interval of 1.59 cM, total length of 1839.75 cM for Pop. 2 with an average interval of 1.65 cM, total length of 1863.00 cM for Pop. 3 with an average interval of 1.50 cM, and total length of 1629.48 cM for Pop. 4 with an average interval of 1.48 cM.

QTL mapping was conducted using the composite-interval mapping method (CIM) of the software Windows QTL cartographer version 2.5 (Zeng 1994). For the CIM, Model 6 of the Zmaoqtl module was used to detect QTL and estimate their effects. The genome was scanned every 2 cM between markers and putative QTL with a window size of 10 cM. The maximum number of cofactors was used to control the genetic background for each trait. Five control markers were identified by forward and backward regression. Empirical threshold levels for declaring QTL significance at the 5% genome-wide type I error level were independently set for each trait by performing 1000 random permutations. The phenotypic variation and additive effect explained by each QTL were estimated from the value expressed by the QTL peaks obtained from the CIM.

### QTL integration and meta-analysis

To integrate the QTL information on the ILAU for the four RIL populations in this study, the four linkage maps were integrated and the number and positions of consensus QTL were estimated by meta-analysis (Goffinet and Gerber 2000; Arcade *et al.* 2004; Chardon *et al.* 2004). The initial QTL identified in the four populations were projected onto the integrated map using their positions and the confidence intervals shared by the four genetic linkage maps. A modified Akaike’s information criterion (AIC) was calculated to select the QTL models with varying numbers of mQTLs, and the model with the lowest AIC value was selected as significant and used to identify the number of mQTLs on each chromosome.

## Results

### Phenotypic performance of the internode length of five parents and four RIL populations

The five parental lines have 5–7 internodes above the uppermost ear, and all five trait values were markedly lower for Yu82, Zong3, and Yu537A than for Yu87-1 and Shen137 (Table 1). For the RIL populations, the trait values exhibited high variability and transgressive segregation, with trait values exceeding the range of the parental lines. All traits were normally distributed in the four RIL populations (Table 1). The variances of genotype were significant at *P* = 0.01, and the variance interactions between genotype and environment were not significant for the measured traits (Table 2).

**Table 1:** Phenotypic performance of the five parental lines and four RIL populations

Population	Parameter	ILFir (cm)	ILSec (cm)	ILThi (cm)	ILFor (cm)	ILFif (cm)
Yu82	Mean ± SD	25.42 ± 1.86	13.74 ± 1.23	12.60 ± 1.33	11.99 ± 1.53	11.21 ± 1.32
Zong3	Mean ± SD	26.44 ± 2.57	13.32 ± 1.43	11.21 ± 1.42	10.87 ± 1.22	10.23 ± 1.42
Yu537	Mean ± SD	25.68 ± 2.45	12.48 ± 1.63	11.50 ± 1.21	10.52 ± 1.33	10.00 ± 1.67
Yu87-1	Mean ± SD	30.14 ± 2.23	14.79 ± 1.45	14.60 ± 1.36	13.75 ± 1.44	12.77 ± 1.42
Shen137	Mean ± SD	29.29 ± 1.89	15.00 ± 1.65	14.57 ± 1.38	14.06 ± 1.32	13.70 ± 1.52
Yu82×Yu87-1	Mean ± SD	26.15 ± 2.27	14.33 ± 1.11	13.97 ± 1.12	13.43 ± 1.14	12.70 ± 1.15
	Range	16.78–39.00	11.47–18.67	10.58–18.44	8.91–17.86	7.92 ± 18.00
	Skewness	0.01	0.40	0.24	0.19	0.18
	Kurtosis	0.57	−0.09	−0.19	−0.23	−0.23
	*h^2^* (%)	79.34	80.09	76.52	81.26	69.86
		70.51–84.87	69.44–82.65	65.83–83.72	66.75–84.01	60.35–78.44
Yu82×Shen137	Mean ± SD	28.00 ± 3.16	13.9 ± 2.11	13.72 ± 2.23	13.80 ± 2.12	12.10 ± 2.12
	Range	20.30–34.09	9.81–17.81	8.74–17.35	8.49–16.59	8.06–16.72
	Skewness	0.07	0.03	0.24	0.19	−0.02
	Kurtosis	0.65	0.57	0.71	−0.25	−0.18
	*h^2^* (%)	83.45	78.79	77.61	78.32	67.97
		72.54–87.46	65.06–82.35	67.38–85.29	65.69–87.52	59.52–79.88
Zong3×Yu87-1	Mean ± SD	26.95 ± 4.25	14.29 ± 2.18	12.88 ± 2.17	12.38 ± 2.18	11.62 ± 2.22
	Range	18.03–37.03	6.50–29.33	7.44—20.67	6.33–20.67	2.50–19.33
	Skewness	0.09	1.02	0.31	0.41	0.21
	Kurtosis	0.62	0.74	0.76	1.03	0.72
	*h^2^* (%)	80.67	82.56	78.63	79.43	68.72
		65.34–86.29	70.03–87.60	60.79–84.32	68.57–86.35	61.87–80.35
Yu537×Shen137	Mean ± SD	25.86 ± 3.42	12.87 ± 2.14	12.15 ± 2.32	11.83 ± 1.89	11.56 ± 2.01
	Range	18.72–35.00	9.44–21.50	5.17–19.78	5.92–17.50	6.94–20.50
	Skewness	0.27	0.39	0.59	0.37	1.00
	Kurtosis	1.40	0.60	0.92	0.83	0.87
	*h^2^* (%)	82.59	76.83	78.54	77.05	65.73
		71.30–87.45	65.09–82.44	69.72–84.69	64.33–82.18	54.62–80.33

ILFir, the first internode length above the uppermost ear; ILSec, the second internode length above the uppermost ear; ILThi, the third internode length above the uppermost ear; ILFor, the fourth internode length above the uppermost ear; ILFif, the fifth internode length above the uppermost ear.

**Table 2:** Analysis of variance for the studied traits in four RIL populations (F-test)

Population	Variation Source	ILFir	ILSec	ILThi	ILFor	ILFif
Yu82×Shen137	Family	4.19^†^	4.99^†^	3.45^†^	4.18^†^	4.00^†^
	Location	1.08	1.80	1.19	1.18	1.23
	Family×location	0.61	0.36	0.25	0.63	0.63
Yu82×Yu87-1	Family	9.80^†^	7.65^†^	8.39^†^	9.30^†^	8.23^†^
	Location	0.98	1.66	1.83	1.90	1.93
	Family×location	0.95	1.22	1.12	1.04	1.11
Zong3×Yu87-1	Family	2.25^†^	3.27^†^	2.40^†^	3.22^†^	3.00^†^
	Location	0.86	1.04	1.01	1.19	1.22
	Family×location	0.82	0.93	0.86	0.67	0.62
Yu537×Shen137	Family	3.35^†^	2.37^†^	3.68^†^	4.14^†^	3.66^†^
	Location	0.86	1.04	1.01	1.19	1.22
	Family×location	0.82	0.93	0.86	0.67	0.62

ILFir, the first internode length above the uppermost ear; ILSec, the second internode length above the uppermost ear; ILThi, the third internode length above the uppermost ear; the fourth internode length above the uppermost ear; ILFif, the fifth internode length above the uppermost ear.

† Significant at *P* = 0.01.

The broad-sense heritability (*h^2^*) for ILAU was high and ranged from 65.73% to 83.45% in the four RIL populations (Table 1). The phenotypic correlations among the five traits were calculated based on the combined data for the three environments (Table 3). The correlation coefficients between internode lengths among positions were significant (*P* = 0.01) and ranged from 0.27 to 0.85 in four RIL populations.

**Table 3:** Correlation coefficients of ILAU in the four RIL populations

Population	Trait	ILSec	ILThi	ILFor	ILFif
Yu82×Yu87-1	ILFir	0.51^†^	0.52^†^	0.43^†^	0.30^†^
	ILSec		0.80^†^	0.67^†^	0.51^†^
	ILThi			0.85^†^	0.67^†^
	ILFor				0.82^†^
Yu82×Shen137	ILFir	0.28^†^	0.29^†^	0.25^†^	0.36^†^
	ILSec		0.40^†^	0.58^†^	0.35^†^
	ILThi			0.42^†^	0.53^†^
	ILFor				0.61^†^
Zong3×Yu87-1	ILFir	0.39^†^	0.41^†^	0.36^†^	0.27^†^
	ILSec		0.71^†^	0.63^†^	0.54^†^
	ILThi			0.76^†^	0.67^†^
	ILFor				0.80^†^
Yu537×Shen137	ILFir	0.52^†^	0.57^†^	0.48^†^	0.36^†^
	ILSec		0.78^†^	0.76^†^	0.60^†^
	ILThi			0.84^†^	0.66^†^
	ILFor				0.75^†^

ILFir, the first internode length above the uppermost ear; ILSec, the second internode length above the uppermost ear; ILThi, the third internode length above the uppermost ear; ILFor, the fourth internode length above the uppermost ear; ILFif, the fifth internode length above the uppermost ear.

† Significant at *P* = 0.01.

### QTL identification for leaf width in the four populations

Seventy QTL for ILFir, ILSec, ILThi, ILFor, and ILFif were mapped to all maize chromosomes in the four RIL populations, with 16 QTL in Pop. 1, 14 QTL in Pop. 2, 25 QTL in Pop. 3, and 15 QTL in Pop. 4 (Table 4). The contributions to phenotypic variation for a single QTL ranged from 5.36% to 26.85%, with 27 QTL >10%.

**Table 4:** QTL detected for internode length above the uppermost ear in the four recombinant inbred line (RIL) populations across three environments

Trait	QTL	Chr	Ph (bp)	Marker Interval	LOD	R^2^ (%)	A
**Yu82×Yu87-1**							
ILFir	q1ILFir1	1	54,453,173–62,011,928	PZE-101071898–PZE-101071162	2.80	6.88	0.70
	q1ILFir4	4	3,895,258–4,086,729	PZE-104005295–PZE-104005482	3.45	6.08	−0.77
	q1ILFir5-1	5	163,198,379–166,411,388	PZE-105107181–PZE-105109134	8.47	16.65	−1.41
	q1ILFir5-2	5	19,568,7817–196,106,672	PZE-105141173–PZE-105141465	4.88	9.44	−0.54
ILSec	q1ILSec4	4	3,895,258–4,086,729	PZE-104005295–PZE-104005482	3.25	7.68	−0.46
	q1ILSec5-1	5	18,667,073–20,693,395	SYN1601–SYN20306	6.05	15.68	−0.55
	q1ILSec5-2	5	195,687,817–196,106,672	PZE-105141173–PZE-105141465	4.88	9.44	−0.54
ILThi	q1ILThi1	1	49,515,795–54,453,173	PZE-101066291–PZE-101071898	2.71	7.10	0.65
	q1ILThi4	4	3,895,258–4,086,729	PZE-104005295–PZE-104005482	2.90	5.36	−0.40
	q1ILThi5	5	194,848,500–196,987,610	PZE-105141007–PZE-105142709	6.56	16.24	−0.88
	q1ILThi7	7	151,536,567–156,648,103	PZE-107105855–PZE-107107154	3.79	7.22	0.47
ILFor	q1ILFor4	4	3,895,258–4,086,729	PZE-104005295–PZE-104005482	3.33	6.87	−0.46
	q1ILFor5	5	194,848,500–196,987,610	PZE-105141007–PZE-105142709	6.62	14.69	−0.69
	q1ILFor7	7	9,799,796–12,523,342	PZE-107013546–PZE-107015480	2.57	6.69	−0.62
ILFif	q1ILFif5	5	194,848,500–196,987,610	PZE-105141007–PZE-105142709	2.93	7.45	−0.55
	q1ILFif7	7	9,799,796–12,523,342	PZE-107013546–PZE-107015480	2.65	6.67	−0.52
**Yu82×Shen137**							
ILFir	q2ILFir7	7	1,979,634–4,709,247	PZE-107002330–SYN20419	10.83	26.85	−1.87
	q2ILFir9	9	101,408,206–104,373,477	PZE-109062229–PZB02480.1	4.55	11.21	0.63
	q2ILFir10	10	142,484,530–143,114,064	PZE-110096735–SYN12154	3.10	8.18	−0.76
ILSec	q2ILSec5	5	189,537,160–193,310,292	PZE-105133858–PZE-105138182	3.95	9.49	0.50
	q2ILSec9	9	133,745,032–135,152,250	PZE-109090207–PZE-109086476	2.81	7.83	0.42
	q2ILSec10	10	123,635,404–124,526,091	SYN15051–PZE-110068110	4.08	10.15	0.52
ILThi	q2ILThi9	9	133,745,032–135,152,250	PZE-109090207–PZE-109086476	2.85	7.54	0.43
ILFor	q2ILFor3	3	19,654,756–20,022,149	PZE-103026528–PZE-103027544	3.67	8.86	0.57
	q2ILFor6	6	155,609,243–156,368,157	PZE-106104150–PZE-106105801	3.07	7.88	0.53
	q2ILFor9	9	133,745,032–135,152,250	PZE-109090207–PZE-109086476	3.08	8.35	0.52
ILFif	q2ILFif1	1	93,613,551–97,628,380	PZE-101099263–PZE-101101518	3.34	7.75	0.82
	q2ILFif6	6	155,609,243–156,368,157	PZE-106104150–PZE-106105801	2.87	8.18	0.57
	q2ILFif7	7	1,979,634–4,709,247	PZE-107002330–SYN20419	10.33	25.63	−1.64
	q2ILFif9		144,508,752–141,768,418	PZE-109099670–PZE-109102157	3.08	8.10	0.65
**Zong3×Yu87-1**							
ILFir	q3ILFir1	1	189,522,638–19,4527,599	PZE-101146525–PZE-101150712	5.89	14.41	−1.44
	q3ILFir3	3	169,008,365–175,554,472	PZE-103110355–PZE-103115618	3.15	6.82	−0.97
	q3ILFir5-1	5	63,405,163–80,802,797	SYN26864–PZE-105073970	4.32	9.13	−1.15
	q3ILFir5-2	5	178,630,838–184,840,977	SYN38466–PZE-105128589	3.08	9.77	1.23
	q3ILFir10	10	128,463,363–130,267,668	PZE-110072258–PZE-110073576	4.73	9.87	1.17
ILSec	q3ILSec3	3	169,008,365–175,554,472	PZE-103110355–PZE-103115618	3.28	8.14	−0.60
	q3ILSec5-1	5	63,405,163–80,802,797	SYN26864–PZE-105073970	3.19	7.36	0.58
	q3ILSec5-2	5	200,776,837–201,018,062	PZE-105150110–PZE-105150391	3.44	10.39	0.75
	q3ILSec9	9	135,296,920–140,436,979	PZE-109092637–PZE-109094331	3.15	8.85	−0.58
	q3ILSec10	10	122,764,555–123,364,763	PZE-110066732–PZE-110067110	2.70	7.53	0.53
ILThi	q3ILThi2	2	17,397,718–17,903,733	SYN18094–PZE-102037260	2.58	6.10	0.54
	q3ILThi3	3	169,008,365–175,554,472	PZE-103110355–PZE-103115618	4.40	11.54	−0.73
	q3ILThi5-1	5	80,223,529–80,802,797	PZE-105074181–PZE-105073970	4.13	10.38	−0.71
	q3ILThi5-2	5	179,626,240–184,744,673	PZE-105123635–PZE-105128217	3.62	10.93	0.89
	q3ILThi9	9	116,763,031–127,820,710	PZE-109074509–PZE-109079845	3.61	8.86	−0.65
ILFor	q3ILFor1	1	220,106,564–219,701,709	SYN2411–SYN19847	4.25	7.48	−0.92
	q3ILFor2	2	17,397,718–19,905,537	SYN18094–PZE-102039914	3.25	9.27	0.91
	q3ILFor3	3	169,008,365–175,554,472	PZE-103110355–PZE-103115618	5.79	17.45	−1.22
	q3ILFor5-1	5	80,223,529–80,802,797	PZE-105074181–PZE-105073970	5.08	14.04	−1.21
	q3ILFor5-2	5	17,962,6240–184,744,673	PZE-105123635–PZE-105128217	3.29	6.92	0.70
	q3ILFor9	9	117,976,872–127,820,710	PZE-109075481–PZE-109079845	4.58	11.15	−0.90
ILFif	q3ILFif2	2	24,585,454–34,305,177	PZE-102047066–PZE-102056295	3.19	8.61	1.01
	q3ILFif5	5	179,626,240–184,744,673	PZE-105123635–PZE-105128217	2.99	7.04	0.82
	q3ILFif8	8	149,193,811–155,571,435	PZE-108092173–PZA03698.1	3.94	11.13	−1.00
	q3ILFif9	9	121,822,604–135,146,198	PZE-109078269–PZE-109086475	6.38	16.76	−1.15
**Yu537×Shen137**							
ILFir	q4ILFir3-1	3	8,270,856–9,886,045	PZE-103015388–SYN36244	3.54	12.01	−0.88
	q4ILFir3-2	3	19,654,756–20,022,149	PZE-103026528–PZE-103027544	2.85	8.71	−0.72
ILSec	q4ILSec2-1	2	182,674,225–190,259,082	PZE-102136708–PZE-102137972	3.79	11.34	0.47
	q4ILSec2-2	2	217,295,349–220,397,345	SYN27033–PZA01991.3	3.76	14.58	0.79
	q4ILSec6	6	113,705,211–128,207,560	PZA00182.4–PZE-106072609	2.53	8.10	−0.40
ILThi	q4ILThi2	2	182,674,225–190,259,082	PZE-102136708–PZE-102137972	4.66	14.06	0.60
	q4ILThi5	5	179,270,149–180,440,398	PZE-105122012–PZE-105123697	3.70	10.88	0.71
ILFor	q4ILFor2-1	2	182,674,225–190,259,082	PZE-102136708–PZE-102137972	7.32	18.36	0.75
	q4ILFor2-2	2	206,357,621–210,816,696	PZE-102165687–PZE-102168198	2.70	6.71	−0.45
	q4ILFor6-1	6	82,616,403–86,257,528	PZE-106036132–PZE-106038001	2.82	6.46	0.45
	q4ILFor6-2	6	113,738,213–11,4026,921	PZE-106062631–PZE-106062790	3.92	9.61	−0.54
ILFif	q4ILFif2	2	182,674,225–190,259,082	PZE-102136708–PZE-102137972	4.32	13.10	0.78
	q4ILFif3	3	124,139,760–132,620,186	PZE-103077185–PZE-103082258	4.05	13.83	−0.84
	q4ILFif4	4	175,440,362–243,222,942	PZE-104099080–PZE-104152506	3.09	10.36	−0.77
	q4ILFif6	6	161,570,136–161,844,331	PZE-106115356–PZE-106116148	2.64	7.06	−0.64

A, additive effect; ILFir, the first internode length above the uppermost ear; ILSec, the second internode length above the uppermost ear; ILThi, the third internode length above the uppermost ear; ILFor, the fourth internode length above the uppermost ear; ILFif, the fifth internode length above the uppermost ear. Additive effect: positive values indicate that Yu82, Zong3, or Yu537 carries the allele for an increase in the traits, whereas negative values indicate that Shen137 or Yu87-1 carries the allele for an increase in the trait value.

The 16 QTL affecting internode lengths above the uppermost ear were located on chromosomes 1, 4, 5, and 7 in Pop. 1, with four QTL for ILFir, three QTL for ILSec, four QTL for ILThi, three QTL for ILFor, and two QTL for ILFif. The contributions of the QTL to phenotypic variation ranged from 5.36% to 16.65%, with four QTL >10%. All positive alleles with the exception of q1ILFir1, q1ILThi1, and q1ILThi7 were contributed by Yu87-1. The QTL q1ILFir4, q1ILSec4, q1ILThi4, and q1ILFor4 were located in the same region between PZE-104005295 and PZE-104005482. The QTL q1ILFir5-2, q1ILSec5-2, q1ILThi5, q1ILFor5, and q1ILFif5 were located in the same region between PZE-105141173 and PZE-1051414651.

The 14 QTL in Pop. 2 were located on all chromosomes except chromosomes 2, 4, and 8. Among the detected QTL, three were associated with ILFir, three were associated with ILSec, one was associated with ILThi, three were associated with ILFor, and four were associated with ILFif. Individual QTL explained 7.54%–26.85% of the total phenotypic variance. Eight positive alleles of the 14 QTL were derived from Shen137 and contributed to increased internode lengths above the uppermost ear. The QTL q2ILFir7 and q2ILFif7 were detected at the same marker interval, PZE-107002330–SYN20419, with two QTL accounting for >20% of the phenotypic variation. The QTL q2ILSec9, q2ILThi9, and q2ILFor9 were detected at the same marker interval, PZE-109090207–PZE-109086476. The QTL q2ILFor6 and q2ILFif6 were detected at the same marker interval, PZE-106104150–PZE-106105801.

Twenty-five QTL in Pop. 3 were associated with internode lengths above the uppermost ear, including five QTL for ILFir, five QTL for ILSec, five QTL for ILThi, six QTL for ILFor, and four QTL for ILFif. These QTL were distributed across the entire genome with the exception of chromosomes 4, 6, and 7, and they accounted for 6.10%–16.76% of the phenotypic variation. Positive alleles of 14 QTL were derived from Yu87-1 and contributed to increased internode length values above the uppermost ear. The QTL q3ILFir3, q3ILSec3, q3ILThi3, and q3ILFor3 were detected in the same region between PZE-103110355 and PZE-103115618, with two QTL accounting for >10% of the phenotypic variation. The QTL q3ILFir5-2, q3ILThi5-2, q3ILFor5-2, and q3ILFif5 were detected in the same region between PZE-105123635 and PZE-105128217. The QTL q3ILThi9 and q3ILFor9 were located in the same chromosomal regions between PZE-109075481 and PZE-109079845 and accounted for 8.86% and 11.15% of the total phenotypic variation, respectively.

The 15 QTL associated with internode lengths above the uppermost ear in Pop. 4 were identified on chromosomes 2, 3, 4, 5, and 6, with two QTL for ILFir, three QTL for ILSec, two QTL for ILThi, four QTL for ILFor, and four QTL for ILFif, accounting for 6.71%–18.36% of the phenotypic variance. The positive alleles of eight QTL were derived from Shen137 and contributed to increases in the trait values. The QTL q4ILSec2-1, q4ILThi2, q4ILFor2-1, and q4ILFif2 were identified in the same region between PZE-102136708 and PZE-102137972, with all QTL accounting for >10% of the phenotypic variation. The QTL q4ILSec6 and q4ILFor6-2 were detected in the same region between PZE-106062631 and PZE-106062790.

### Genetic map integration and mQTL analysis

The mQTL analysis was a superior method for identifying true and consistent QTL for the measured traits from the four RIL populations, and the genetic map and initial QTL were integrated by meta-analysis. The integrated genetic map included 2439 SNP markers and was 1933.74 cM long, with an average of 0.79 cM between markers based on the four populations. After integration, 46 initial QTL were captured by 14 mQTLs located on all chromosomes except for chromosome 8 (Table 5). The mean phenotypic variation of the initial QTL synthesized into the corresponding mQTLs was 5.36%–26.85%, with 17 QTL >10%. One mQTL included an average of 3.29 initial QTL, with a range of 2 to 11 for 2 to 4 traits.

**Table 5:** The mQTLs for internode length above the uppermost ear in four sets of recombinant inbred line (RIL) populations

mQTL	Chr	Flanking Markers	Physical Intervals (pb)	No. of QTL	Integrated QTL	Candidate Gene/Previous Studies About Plant Height in Maize
mQTL1	1	PZE-101071898–PZE-101071162	54,453,173–62,011,928	2	q1ILFir1, q1ILThi1	Peiffer *et al.* (2014)
mQTL2-1	2	SYN18094–PZE-102037260	17,397,718–17,903,733	2	q3ILThi2, q3ILFor2	
mQTL2-2	2	PZE-102136708–PZE-102137972	182,674,225–190,259,082	4	q4ILSec2-1, q4ILThi2, q4ILFor2-1, q4ILFif2	GRMZM2G001977
mQTL3-1	3	PZE-103026528–PZE-103027544	19,654,756–20,022,149	2	q4ILFir3-2, q2ILFor3	
mQTL3-2	3	PZE-103110355–PZE-103115618	169,008,365–175,554,472	4	q3ILSec3, q3ILFir3, q3ILThi3, q3ILFor3	Ajmone-Marsan *et al.* 1994; Kozumplik *et al.* 1996; Lübberstedt *et al.* 1997; Melchinger *et al.* 1998; Tang *et al.* 2007; Elisabetta *et al.* 2007; Gonzalo *et al.* 2010
mQTL4	4	PZE-104005295–PZE-104005482	3,895,258–4,086,729	4	q1ILFir4, q1ILSec4, q1ILThi4, q1ILFor4	
mQTL5-1	5	PZE-105074181–PZE-105073970	80,223,529–80,802,797	4	q3ILFir5-1, q3ILSec5-1, q3ILThi5-1, q3ILFor5-1	
mQTL5-2	5	PZE-105128217–PZE-105141007	184,744,673–194,848,500	11	q1ILFir5-2, q1ILSec5-2, q1ILThi5, q1ILFor5, q1ILFif5, q2ILSec5, q3ILFir5-2, q3ILThi5-2, q3ILFor5-2, q3ILFif5, q4ILThi5	Ajmone-Marsan *et al.* 1994; Lübberstedt *et al.* 1997; Tang *et al.* 2007; Gonzalo *et al.* 2010
mQTL6-1	6	PZE-106062631–PZE-106072609	113,738,213–128,207,560	2	q4ILSec6, q4ILFor6-2	GRMZM2G097132
mQTL6-2	6	PZE-106104150–PZE-106105801	155,609,243–156,368,157	2	q2ILFor6, q2ILFif6	
mQTL7-1	7	PZE-107002330–SYN20419	1,979,634–4,709,247	2	q2ILFir7, q2ILFif7	
mQTL7-2	7	PZE-107013546–PZE-107015480	9,799,796–12,523,342	2	q1ILFor7, q1ILFif7	GRMZM2G115357
mQTL9	9	PZE-109079845–PZE-109090207	127,820,710–133,745,032	6	q2ILSec9, q2ILThi9, q2ILFor9, q3ILThi9, q3ILFor9, q3ILFif9	Peiffer *et al.* (2014)
mQTL10	10	PZE-110066732–SYN15051	122,764,555–123,635,404	2	q2ILSec10, q3ILSec10	

The initial QTL included in mQTL5-2 with four QTL >10% were identified for five internode lengths above the uppermost ear in two populations; the initial QTL in mQTL9 with two QTL >10% were demonstrated for three traits in two populations; the initial QTL in mQTL3-1 were identified for one trait in two populations; the initial QTL in mQTL2-2, mQTL3-2, mQTL4, and mQTL5-1 were identified for four traits in one population; and the QTL identified in the remaining mQTLs were identified for two traits in one population.

## Discussion

Increasing grain yield per unit area is one of most important objectives of maize breeding. In recent decades, numerous approaches (improved photosynthesis, nutrient use efficiency, tolerance to biotic and abiotic stress) have been proposed to increase grain yield per unit area (Bertin and Gallais 2001; Tuberosa *et al.* 2002; Hund *et al.* 2004; Grance *et al.* 1987; Louwerse and Zweerde 1997). The effect of changes in growth processes and plant traits on yield at the canopy level is unclear (Zhu *et al.* 2012; Evans 2013). However, canopy morphology is an important and complicated agronomic trait, and research has focused on leaf area, leaf angle, leaf length, tassel morphology, and QTL mapping (Mickelson *et al.* 2002; Ku *et al.* 2010, 2012; Li *et al.* 2008; Tian *et al.* 2011). The QTL responsible for ILAU have not been identified. In this study, the QTL for each ILAU were mapped using four sets of RIL populations evaluated in three environments, which resulted in the identification of 70 QTL: 16 QTL in Pop. 1, 14 QTL in Pop. 2, 25 QTL in Pop. 3, and 15 QTL in Pop. 4 (Table 4). Individual effects ranged from 5.36% to 26.85% of the phenotypic variation, with 27 QTL contributing >10%. Forty-six initial QTL were included in 14 mQTLs by meta-analysis, and 17 of the 27 initial QTL with R^2^ >10% were included in 7 mQTLs. These results could provide useful information for marker-assisted selection (MAS) to improve canopy architecture.

### Comparison of the mapping results across five traits measured in the four RIL populations

We verified three common QTL located in the same or similar chromosome regions, one for ILAU at all positions in bin 5.06, one for three measured traits in bin 9.06, and one for ILSec in bin 10.04 across two RIL populations (Table 5). Although four common QTL were identified for four measured traits in one population, six common QTL were identified for two measured traits in one population, and additional QTL were identified for one measured trait in different populations. The position consistency of the QTL demonstrated that all internode lengths above the uppermost ear may be affected by one or several of the same QTL. In addition, the position-specific QTL demonstrated that internode length at different positions above the uppermost ear may be regulated by many different QTL. In terms of different populations, one QTL for five measured traits in bin 5.06, one QTL for three measured traits in bin 9.06, and one QTL for one measured trait in bin 10.04 were located across two RIL populations. These QTL were consistent across the two populations and merit further study via the construction of isogenic lines (NILs). Although the two populations exhibited certain similarities because they shared a parental line, population-specific QTL (*e.g.*, QTL in bin 2.07, QTL in bin 4.01, QTL in bin 5.03) were also identified. Population-specific QTL were attributed to differences in the genetic background between the two populations because they shared only one common parental line.

### Synthesis of initial QTL for five traits across four populations and comparison of QTL related to plant height in previous studies

To determine if the loci identified in different QTL mapping studies are identical, the chromosomal regions of a common subset of markers were compared across different studies or indirectly compared by evaluating each mapping population according to a reference map (Tuberosa *et al.* 2002). mQTL analyses are a superior method for combining data from independent studies to identify consensus QTL and for revealing genetic correlations among traits. In maize, mQTLs for drought tolerance, flowering, grain yield components, ear rot resistance, leaf architecture, and silage quality have been reported (Danan *et al.* 2011; Truntzler *et al.* 2010; Hao *et al.* 2010; Chardon *et al.* 2004; Li *et al.* 2011; Xiang *et al.* 2012, 2010; Ku *et al.* 2012). In the present study, we identified 14 mQTLs from a total of 70 QTL in the four populations for each ILAU using mQTL analyses, and 62.96% of the QTL showed R^2^ <10%. mQTL5-2 included 11 initial QTL for internode length at five positions from two populations that explained 6.92%–16.24% of the phenotypic variation. mQTL9 included six QTL for internode length at three positions from two populations that explained 7.54%–16.76% of the phenotypic variation. mQTL10 included two QTL for internode length at one position from two populations that explained 10.15% and 7.53% of the phenotypic variation. mQTL2-2, mQTL3-2, and mQTL5-1 included four QTL for internode length at four positions from one population, with at least two initial QTL with R^2^ <10%. These mQTLs, particularly mQTL2-2, mQTL5-1, and mQTL9, may merit further analysis via the construction of secondary mapping populations, such as fine mapping, MAS, and map-based cloning. Studies of mQTL2-2, mQTL5-1, and mQTL9 are currently underway in our laboratory. In short, the analysis of each ILAU at the molecular level revealed mQTLs associated with these traits in the four populations, potentially enabling their simultaneous improvement through MAS.

Plant height was determined by internode length and internode number. Plant height has been widely studied, although internode length has not been researched. In studies on QTL of plant height, numerous common QTL have been identified across different populations and different environments (Table 5). These mQTLs include shared alleles identified in the present study and studies on plant height (Peiffer *et al.* 2014) between PZE-101071898 and PZE-101071162 on chromosome 1 for ILAU; shared alleles identified in the present study and studies on plant height (Zhang 2010; Ajmone-Marsan *et al.* 1994; Kozumplik *et al.* 1996; Lübberstedt *et al.* 1997; Melchinger *et al.* 1998; Tang *et al.* 2007; Elisabetta *et al.* 2007; Gonzalo *et al.* 2010) between PZE-103110355 and PZE-103115618 on chromosome 3 for ILAU as well as for other generations (RIL and F2:3 generations); shared alleles identified in the present study and studies on plant height (Ajmone-Marsan *et al.* 1994; Lübberstedt *et al.* 1997; Tang *et al.* 2007; Gonzalo *et al.* 2010) between PZE-105128217 and PZE-105141007 on chromosome 5 detected in the present study for ILAU; and shared alleles identified in the present study and studies on plant height (Peiffer *et al.* 2014) between PZE-109079845 and PZE-109090207 on chromosome 9 for ILAU. These results showed that mQTLs affected plant height by acting on internode length. In addition, ILAU-specific mQTLs (*e.g.*, mQTL3-1, mQTL 2-1, etc.) were also identified.

### Associations with genes in the hormonal pathways of plant height

The studies showed that plant height was affected by plant hormones and other factors that affect cell division, cell elongation, or both (Wang and Li 2008; Komorisono *et al.* 2005). More than 100 genes/loci affect plant height through gibberellin, brassinosteroid, and auxin signaling, transport and synthesis pathways, as well as through cell proliferation (Multani *et al.* 2003; Komorisono *et al.* 2005). To further assess the genetic basis of ILAU variation, the association between mQTLs and genes known to be involved in plant height in Arabidopsis, rice, and maize were investigated through a bioinformatics approach in maize. Three candidate genes controlling plant height were located in three mQTL intervals for ILAU (Table 5). GRMZM2G001977 was located in the mQTL2-2 interval on chromosome 2. The candidate gene is involved in the GA-signaling pathway and putative gibberellin receptor GID1L2 (Alexandrov *et al.* 2009). The gene affects stem growth and the autonomous flowering time pathway (Zhang *et al.* 2012; Griffiths *et al.* 2006). GRMZM2G097132 was mapped to the mQTL6-1 interval on chromosome 6, and the gene belongs to the 2OG-Fe (II) oxygenase superfamily and was 2-oxoglutarate-dependent dioxygenase, which catalyzes subsequent reactions in the GA biosynthesis pathway (Hedden and Phillips 2000). A loss of function in this gene in plants can generate a dwarf phenotype in Arabidopsis. GRMZM2G115357 was mapped to the mQTL7-2 interval on chromosome 7, and the gene is a member of the auxin-induced Aux/IAA family (SHY2/IAA3). A gain-of-function mutation in SHY2/IAA3 causes enlarged cotyledons, short hypocotyls, and altered auxin-regulated root development in Arabidopsis. Therefore, the gene causes pleiotropic auxin-related phenotypes, which indicates that the Aux/IAA gene plays a central role in auxin signaling (Reed 2001). The consistency of the mQTLs and candidate genes identified in this study provided valuable information that was used to fine-map and determine quantitative trait genes and reveal the molecular mechanisms responsible for ILAU.
